# Human Exteroception during Object Handling with an Upper Limb Exoskeleton

**DOI:** 10.3390/s23115158

**Published:** 2023-05-29

**Authors:** Dorine Arcangeli, Océane Dubois, Agnès Roby-Brami, Sylvain Famié, Giovanni de Marco, Gabriel Arnold, Nathanaël Jarrassé, Ross Parry

**Affiliations:** 1 LINP2, UPL, UFR STAPS, Université Paris Nanterre, 200 Avenue de la République, 92001 Nanterre, France; 2 CAYLAR, 14 Avenue du Québec, 91140 Villebonne sur Yvette, France; 3 ISIR, Sorbonne University, CNRS UMR 7222, ERL INSERM U 1150, 75005 Paris, France

**Keywords:** human-exoskeleton interaction, exteroception, dynamic touch, tool use, smart perceptual systems

## Abstract

Upper limb exoskeletons may confer significant mechanical advantages across a range of tasks. The potential consequences of the exoskeleton upon the user’s sensorimotor capacities however, remain poorly understood. The purpose of this study was to examine how the physical coupling of the user’s arm to an upper limb exoskeleton influenced the perception of handheld objects. In the experimental protocol, participants were required to estimate the length of a series of bars held in their dominant right hand, in the absence of visual feedback. Their performance in conditions with an exoskeleton fixed to the forearm and upper arm was compared to conditions without the upper limb exoskeleton. Experiment 1 was designed to verify the effects of attaching an exoskeleton to the upper limb, with object handling limited to rotations of the wrist only. Experiment 2 was designed to verify the effects of the structure, and its mass, with combined movements of the wrist, elbow, and shoulder. Statistical analysis indicated that movements performed with the exoskeleton did not significantly affect perception of the handheld object in experiment 1 (BF_01_ = 2.3) or experiment 2 (BF_01_ = 4.3). These findings suggest that while the integration of an exoskeleton complexifies the architecture of the upper limb effector, this does not necessarily impede transmission of the mechanical information required for human exteroception.

## 1. Introduction

Assistive technologies for the upper limb are generally dependent on the use of tactile and kinesthetic feedback in order to facilitate, or correct the user’s movements [1,2]. Robotic exoskeletons are a particular case, in that they involve fixation of a mechanical structure to the user’s body. This enables forces to be transmitted directly from the structure and applied to different segments, thereby influencing various aspects of task performance (e.g., movement trajectory, intersegmental coordination, muscular effort). At the same time, physical assistance provided by this type of technology directly implicates the somatosensory system, most notably via haptic networks composed of neuromuscular and cutaneous mechanoreceptors [3]. These sensory afferents inform the nervous system regarding the disposition of the different segments of the body as well as the surrounding physical environment [4]. 

To date, research on upper limb exoskeletons has focused primarily on human motor performance. An important part of this work has involved the development of control algorithms capable of negating the effects of the structure itself on the movement dynamics of the individual. Referred to as transparency control, these algorithms use feedforward robotic control to determine movement resistance associated with the mass and mechanical transmission of the exoskeleton such that they can compensate for gravity, inertia and friction [5]. Despite this, experimental studies comparing movement performed with exoskeletons using transparency control to movements performed without an exoskeleton highlight differences in electromyographic activity, joint coordination and end-effector displacement [6,7]. These effects may be associated with mechanical consequences inherent to the physical coupling of the upper limb to the exoskeleton, including subtle, but necessary, variations in alignment of the kinematic chains [8]. Even with sophisticated control algorithms, it thus appears likely that the use of upper limb exoskeletons might inherently alter human motor control [9]. 

The extent to which an exoskeleton affects the user’s perceptual capacities is an equally important concern. The ability to effortlessly perceive one’s interactions with their environment underpins the sense of mastery and intuitive control in everyday motor activity [10]. The salience of somatosensory feedback is thus essential for the appropriation of assistive technology [11]. Certain research programs have attempted to use exoskeleton technology as a means to evaluate proprioceptive acuity in healthy and pathological populations. These protocols have generally involved learning and identifying joint positions or trajectories via guided passive movements with the robotic device [12,13]. Again, these approaches are not without their limits, as cutaneous stimulation from fastenings, and bodyweight support within the structure itself, affect reliability of the measures [14]. More importantly, these examples are not representative of the complex exchanges which occur between the user and exoskeleton in the context of functional tasks. 

For exoskeletons to be useful in practical situations, the user needs to effectively sense and feel not only their own body, but also the tools and materials which they employ [15]. Under normal circumstances, a person may perceive an object’s characteristics simply by taking it in their hands. The moment they begin to manipulate that object, a general impression regarding its length, its inclination with respect to the hand, as well as the position of the hand with respect to the object itself begins to emerge through a process referred to as dynamic touch [16]. These particular forms of nonvisual perception are referred to more precisely as exteroception, exproprioception and proexteroception respectively [17]. Dynamic touch is distinct from direct tactile and proprioceptive perception in that the nervous systems appears to exploit the different mechanical effects associated with the movement (e.g., inertia) in order to estimate the distribution of mass in the handheld object [16]. 

While a number of previous studies have examined the role of upper limb exoskeletons on object handling, this work has been principally oriented towards the benefits of an assistive device on the physical demands of the user [2,18,19]. The potential consequences upon those sensory capacities necessary for adaptive tool use behaviors have been largely overlooked. In the present communication, we investigate the effect of an exoskeleton upon the ability to perceive handheld objects through dynamic touch. In effect, fixation of an exoskeleton alone engages touch receptors at its interface with the user’s arm, just as the circumferential attachments might influence the transmission of forces through the upper limb. Moreover, the exoskeleton itself contributes mass additional to that imposed by the handheld object, and would thereby influence the inertial consequences of the upper limb movements. With this in mind, we proposed two separate experiments which examine human exteroception, the nonvisual perception of object length derived through dynamic touch. These experiments compared the perceived length of handheld objects manipulated both with and without an upper limb exoskeleton fixed to the forearm and upper arm of the user. 

## 2. Materials and Methods

### 2.1. Participants

Twelve healthy adult participants (8 male) with an average age of 24 years (SD 3 years, range 22–31 years) and no known neurological or orthopedic conditions were recruited to this study. All had either normal or corrected vision. Each participant was evaluated as being right hand dominant using the Edinburgh Handedness Inventory [20] with an average laterality index of 75 (SD 9, range 65–95). Average grip force of the cohort was 42 kg (SD 7 kg, range 30–50 kg). These results are consistent with normative data for this age group [21]. 

### 2.2. Exoskeleton

**Figure 1:**
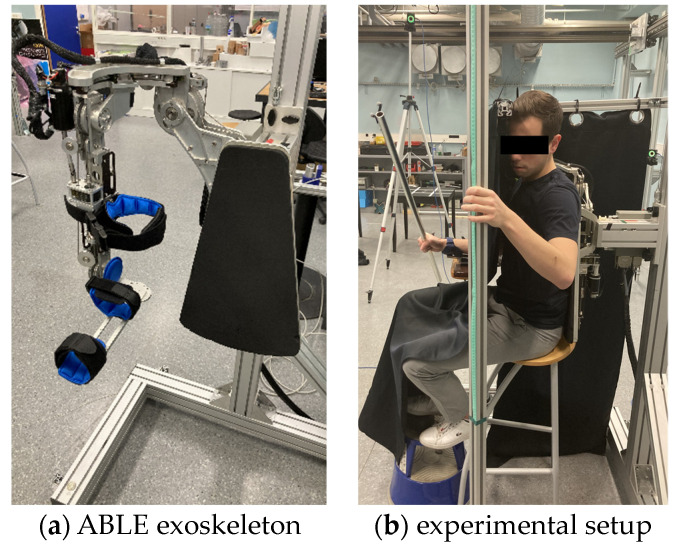
(**a**) The ABLE exoskeleton comprising three rotational axes axes at the shoulder and one at the level of the elbow. Foam and Velcro fixations are used to secure the forearm and upper arm of the user to the structure. (**b**) A participant manipulates a bar with his right hand during an experimental trial. Perceived partial length was indicated using an adjustable marker on a vertical pole positioned to the left of the participant.

An ABLE exoskeleton (Haption, Soulgé-sur-Ouette, France) with four degrees of freedom (DOF) was used for this study [22]. This device comprises three rotational axes at the level of the shoulder (abduction/adduction, flexion/extension, internal/external rotation) and one at the level of the elbow (flexion/extension). The mechanical structure itself limits movement amplitude within physiologically compatible ranges (110° for each rotational axis of the shoulder, 130° for the elbow). It uses foam and Velcro fixations to secure the forearm and upper arm of the user to the structure. The patented screw-cable transmission used by the ABLE exoskeleton limits the overall mass carried by the upper limb (approx. 7 kg) and enhances backdriveability. For the purposes of this experiment, the robotic exoskeleton was used in four different fashions. (1) A static configuration was used to fix the exoskeleton at given amplitudes along the four rotational axes. (2) Rigid control was used to guide the upper limb to a desired configuration. (3) The robotic exoskeleton was attached and deactivated such that it made no active contribution to movement, its mass subject to gravity. (4) The exoskeleton was attached and activated in transparent mode with friction and gravity compensation. In this mode, the robotic controller minimizes resistance to movement associated with mechanical transmission and the mass of the structure itself. Further technical details regarding the exoskeleton are provided in supplementary material S1. Figure 1a provides an image of the ABLE exoskeleton. 

### 2.3. Experimental Setup

The experimental task involved estimating the length of a series of five metal bars. Each bar was cylindrical with a 3 cm diameter. The lengths were 45 cm, 60 cm, 75 cm, 90 cm and 105 cm (mass 0.5 kg–1.2 kg). A small line was inscribed at one third along the length of the bar to indicate the placement of the hand during trials. Participants were seated on a stool with their back resting against the vertical support of the ABLE exoskeleton. Prior to beginning the experiment, the right arm was placed alongside the trunk in a comfortable posture with slight flexion through the shoulder and elbow. This position was verified with a goniometer and recorded as the reference position using the ABLE exoskeleton. 

The experimental task involved moving the bar with the right hand to estimate the length between the position where the bar was held and its extremity. Grasp position was controlled such that the partial lengths (projecting forward from the hand) for the five bars were 30 cm, 40 cm, 50 cm, 60 cm and 70 cm respectively. The participants indicated the perceived (partial) length of the bar by positioning a marker on a vertical pole situated to their left. The same bar was presented three times in a randomized order for each experimental condition. Response time and perceived length were used as the dependent variables to evaluate task performance. All 12 participants completed both experiments described below in a counterbalanced order. Figure 1b provides an image of a participant performing the experimental task. Supplementary Material S2 provides video footage of an experimental trial.

### 2.4. Experiment 1

The first experiment sought to determine if the fixation of an exoskeleton to the user’s arm affected exteroception. Task performance was evaluated in two conditions: (1) exoskeleton attached, and (2) without exoskeleton. In both conditions, the participant’s right arm was placed in the reference position. For the condition with exoskeleton attached, the arm was secured with the habitual fixations, the reference position maintained using the static configuration mode. For the condition without exoskeleton, the segmental components of the ABLE device were completely retracted. Reference position of the participant’s arm was verified using a goniometer and the posture maintained with the aid of a horizontal forearm support. During experiment 1, the participants’ movements were limited to rotations of the wrist (i.e., flexion/extension, radial/ulnar deviation, pronation/supination). This procedure was designed to evaluate the effects of the attachment of the exoskeleton independently of the effects of the exoskeleton’s mass during object handling. 

### 2.5. Experiment 2

The purpose of the second experiment was to verify if human exteroception was affected by displacing the structure of the exoskeleton and its associated mass during object handling. Task performance was evaluated in three conditions: (1) exoskeleton attached deactivated (2) exoskeleton in transparent mode; and (3) without exoskeleton. Habitual fixations were applied during conditions with the exoskeleton, and rigid control was used to return the arm to the reference position at the beginning of each trial. The exoskeleton was fully retracted during the condition without exoskeleton such that upper limb movements were unhindered, while the experimenter verified the reference position at the beginning of each trial. During experiment 2, participants were instructed to use combined rotations of the wrist, elbow and shoulder. In this procedure, object handling with the exoskeleton deactivated served to evaluate the effects of moving the upper limb against resistance due to the mass of the exoskeleton. In the condition using the exoskeleton in transparent mode, exteroception was evaluated during movement where the exoskeleton was attached but actively compensated the resistance associated with its own mass and mechanical transmission. 

### 2.6. Statistical Analysis

Response time and perceived length was averaged for each participant across the three trials with each bar in the different experimental conditions. Pearson correlation coefficients were calculated to verify the overall linear dependency between partial bar length and perceived length for object handling with and without the exoskeleton. Single sample *t*-tests were used to compare mean values of perceived length with partial bar lengths in each of the different conditions. Response time and perceived length variables were then examined using repeated-measures analysis of variance (ANOVA). Factors for the independent variables of partial bar length (30 cm, 40 cm, 50 cm, 60 cm, 70 cm) and exposure to the exoskeleton were included for experiment 1 (exoskeleton attached, without exoskeleton) and experiment 2 (exoskeleton deactivated, exoskeleton transparent, without exoskeleton). Greenhouse–Geisser adjustment was applied to ANOVA models where necessary. Threshold for statistical significance was set at *p* < 0.05 with Holm–Bonferroni corrections used for multiple comparisons in post-hoc testing. Subsequent analyses using Bayesian *t*-tests and repeated measures ANOVA were used to verify the null hypothesis in the absence of significant effects [23,24,25,26]. Results of Bayesian testing were interpreted as providing anecdotal (BF_01_ 1–3), moderate (BF_01_ 3–10) or strong (BF_01_ 10–30) evidence for the null hypothesis [27]. Further details regarding implementation of the statistical analyses are provided in supplementary materials S3 and S4.

## 3. Results

### 3.1. Experiment 1

**Figure 2:**
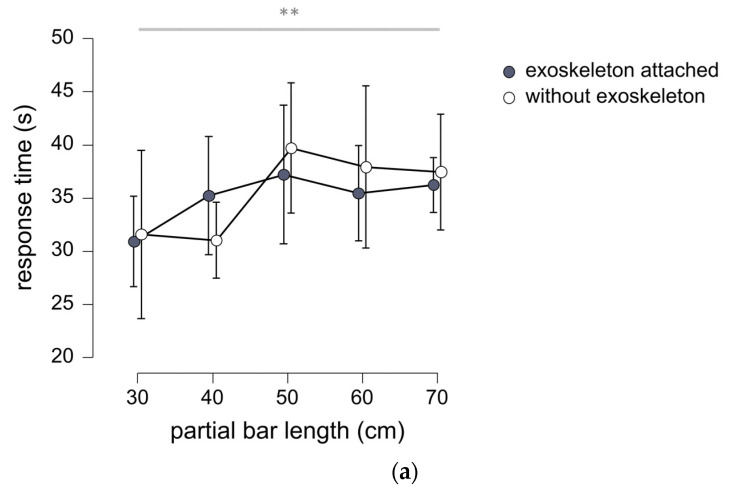

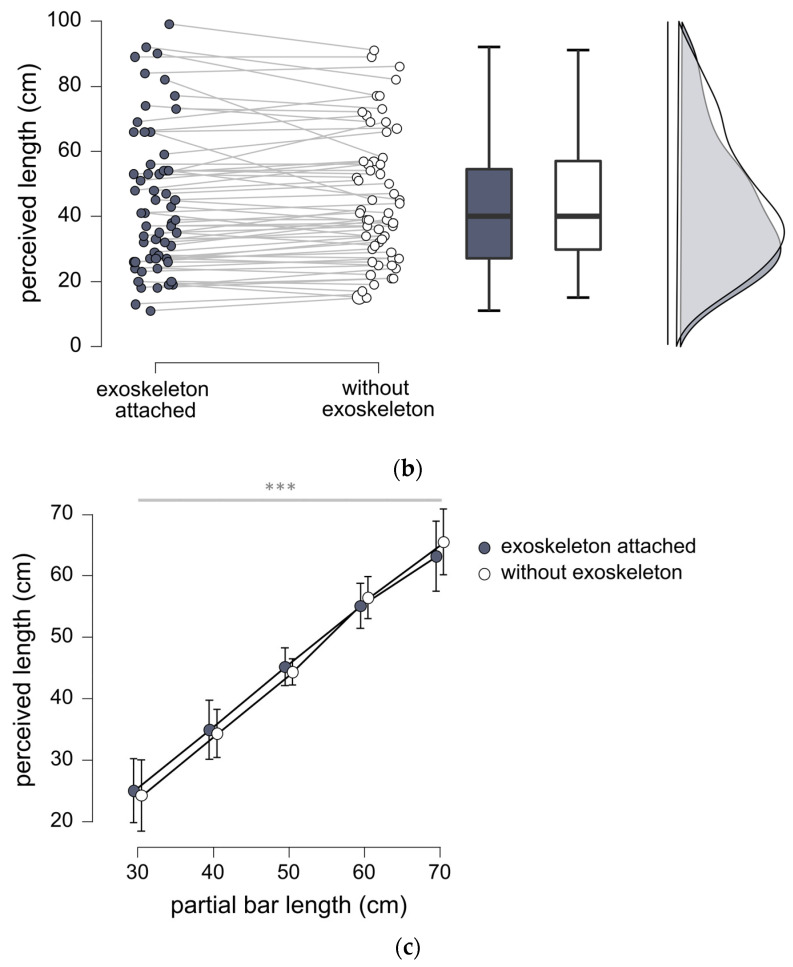
(**a**) Mean response times for different bars manipulated in experiment 1. Error bars represent 95% confidence intervals. Horizontal bar indicates main effect for bar length, double asterisk (**) indicates *p* < 0.01. See text for details on post-hoc testing. (**b**) Raincloud plot for experiment 1 showing distributions for perceived length in the conditions exoskeleton attached and without exoskeleton. (**c**) Mean values of perceived length for the different bars manipulated in experiment 1. Error bars represent 95% confidence intervals. Horizontal bar indicates main effect for bar length, triple asterisk (***) indicates *p* < 0.001. See text for details on post-hoc testing. Note that perceived length and partial bar length both refer to the distance between the hand and the extremity of the handheld object.

Average response time for experiment 1 was 35.0 s (SD 14.1 s) with the exoskeleton attached and 35.5 s (SD 17.3 s) without the exoskeleton. Repeated measures ANOVA confirmed the absence of significant difference between these values while subsequent Bayesian verification of the null hypothesis indicated that the experimental results provided anecdotal evidence that response time was equivalent for the condition with the exoskeleton and the condition without the exoskeleton (BF_01_ = 2.2). Response time was found to vary according to bar length (*p* = 0.002; see Figure 2a). Post-hoc testing indicated that response time for the partial bar length of 30 cm was significantly different from those for the partial bar lengths of 50 cm (*p* = 0.003), 60 cm (*p* = 0.042) and 70 cm (*p* = 0.037), and that response time for the partial bar length of 40 cm was significantly different from the time for the 50 cm partial bar length (*p* = 0.043).

Overall correlation of perceived length and partial bar length was 0.62 (*p* < 0.001) with the exoskeleton attached, and 0.74 (*p* < 0.001) without the exoskeleton. Repeated measures ANOVA confirmed that perceived length of the distance between the hand and the extremity of the bar varied according to the actual values of partial bar length (*p* < 0.001; see Figure 2b,c). Post-hoc testing showed significant differences for perceived length in pairwise comparisons between each bar (*p* = 0.002–*p* < 0.001; refer to statistical tables in supplementary material S3 for details). 

In the condition with the exoskeleton, no significant differences between perceived length and actual length were revealed from single sample *t*-tests. The subsequent Bayesian analysis indicated that the results obtained provided anecdotal evidence that perceived lengths corresponded with the actual lengths (BF_01_ = 1.9–BF_01_ = 2.5; see supplementary material S3). In the condition without exoskeleton, a significant difference between perceived length and actual length was observed for the bar of 45 cm length only (*p* = 0.049). Bayesian testing provided anecdotal evidence in favour of the null hypothesis in all other cases (BF_01_ = 0.9–BF_01_ = 2.6; see supplementary material S3). Repeated measures ANOVA confirmed the absence of a significant difference between length perception in the conditions with and without exoskeleton (see Figure 2b,c). The subsequent Bayesian analysis provided anecdotal evidence that perceived length was equal in the conditions with and without the exoskeleton (BF_01_ = 2.3). 

### 3.2. Experiment 2

**Figure 3:**
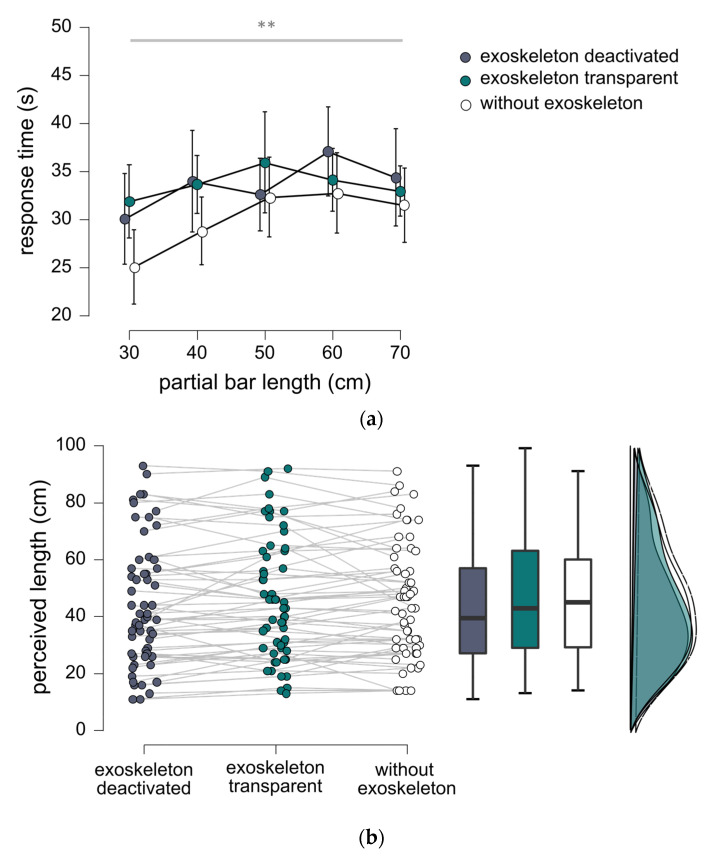

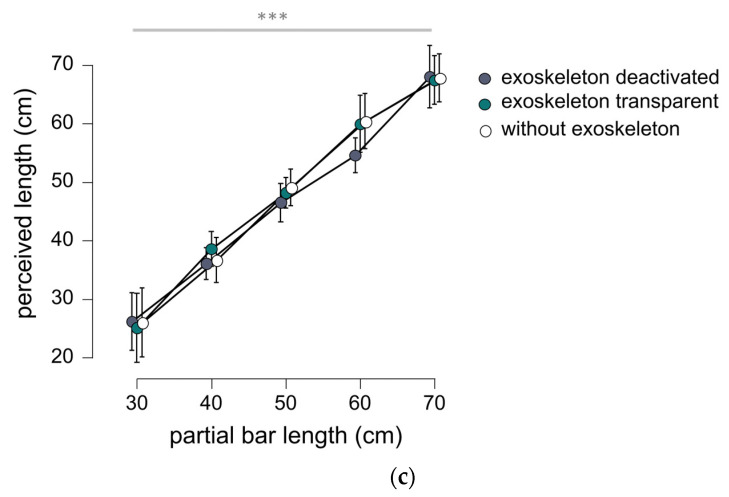
(**a**) Mean response times for different bars manipulated in experiment 2. Error bars represent 95% confidence intervals. Horizontal bar indicates main effect for bar length, double asterisk (**) indicates *p* < 0.01. See text for details on post-hoc testing. (**b**) Raincloud plots for experiment 2 showing distributions for perceived length in the conditions exoskeleton deactivated, exoskeleton transparent and without exoskeleton. (**c**) Mean values of perceived length for the different bars manipulated in experiment 2. Error bars represent 95% confidence intervals. Horizontal bar indicates main effect for bar length, triple asterisk (***) indicates *p* < 0.001. See text for details on post-hoc testing. Note that perceived length and partial bar length both refer to the distance between the hand and the extremity of the handheld object.

Average response time for experiment 2 was 33.6 s (SD 11.1 s) with the exoskeleton deactivated, 33.7 s (SD 10.7 s) with the exoskeleton in transparent mode and 30.1 s (SD 11.4 s) without the exoskeleton. Repeated measures ANOVA indicated that partial bar length had a significant effect upon response time (*p* < 0.001; see Figure 3a). Response time for the 30 cm partial bar length was generally inferior to those for bars of other lengths, with post-hoc testing indicating significant differences with the partial bar lengths of 50 cm (*p* = 0.008), 60 cm (*p* < 0.001) and 70 cm (*p* = 0.033). Exposure to the exoskeleton did not appear to have a significant effect upon response time (BF_01_ = 1.4).

Overall correlation of perceived length and partial bar length was 0.61 (*p* < 0.001) with the exoskeleton deactivated, 0.65 (*p* < 0.001) with the exoskeleton in transparent mode, and 0.67 (*p* < 0.001) without the exoskeleton. Repeated measures ANOVA confirmed that perceived length varied according to the actual partial lengths (*p* < 0.001; see Figure 3b,c). Post-hoc testing showed significant differences for perceived length in pairwise comparisons between all bars (*p* < 0.001). Single sample *t*-tests revealed a significant difference between perceived partial length and actual partial length for the 45 cm bar (i.e., partial length of 30 cm) during object handling with the exoskeleton in transparent mode (*p* = 0.045). Perceived length was found to correspond with actual length in all other conditions (BF_01_ = 1.7–BF_01_ = 3.5; see supplementary materials S4 for details). 

Overall, repeated measures ANOVA indicated that coupling to the exoskeleton did not have a significant effect upon perceived length of the bars (see Figure 3b,c). Bayesian verification of the null hypothesis indicated that these results provided moderate evidence that length perception was equal across the conditions with the exoskeleton deactivated, in transparent mode and without the exoskeleton (BF_01_ = 4.3). 

## 4. Discussion

The present study examined the effects of wearing an upper limb exoskeleton on human exteroception. The experimental protocol involved estimating the partial length of a series of metal bars, which participants manipulated using their dominant right arm. Task performance during conditions with an upper limb exoskeleton was compared to conditions without the exoskeleton in each of the two experiments conducted. The results of this study indicated that mechanical coupling of the arm to an upper limb exoskeleton did not impair the nonvisual perception of handheld objects. This appeared true both when exploratory movements were limited to rotations of the wrist (experiment 1), and when combined rotations of the wrist, elbow and shoulder were mobilized against the mass and inertia of the exoskeleton (experiment 2). In effect, length perception appeared generally equivalent in the conditions with the exoskeleton attached and those without the exoskeleton. Similarly, the time manipulating the bar prior to providing an estimate of partial length was not influenced by exposition to the exoskeleton, although response time differences were observed between bars of different lengths. These findings provide insight regarding the aptitude of the nervous system in appropriating an upper limb exoskeleton, as well as perspectives for human-exoskeleton interaction more generally. 

When attached to the upper limb exoskeleton, the participants’ nonvisual perception of the length between the hand and the extremity of the handheld object was generally comparable with the actual lengths. This observation is consistent with previous studies on exteroception where perceived length is not a perfect match, but within a marginal tolerance of actual length [17]. In essence, exteroception denotes the ability to sense the dimensions of things which are attached to the body. Like other forms of dynamic touch, the muscular effort to displace that attachment entails time-varying deformation of muscles and connective tissues, which in turn stimulate embedded mechanoreceptors [17]. Use of an exoskeleton brings about both local compression of these soft tissues where it is fixed to the human arm, as well as the potentially broader effects of its mass and the consequent resistance to the initiated movement. The particularity of the present study is that participants successfully perceived the dimensions of a handheld bodily attachment (i.e., bar) independently of another bodily attachment fixed at two points on their arm (i.e., exoskeleton). These findings suggest that the nervous system dissociates the mechanical consequences attributable to the handheld object from those attributable to the upper limb exoskeleton during exploratory movement. 

The haptic networks supporting dynamic touch have previously been described as smart perceptual systems. This premise suggests that the perceptual instrument is capable of extracting complex dynamical variables (e.g., inertia) using the physiological and anatomical structures at its disposition [17,28]. The fact that exteroception was not negatively affected by the presence of the exoskeleton would certainly support this perspective. For instance, previous studies emphasise the flexible versatility of dynamic touch, as the same wielding or probing movements are used to extract different features (partial length, whole length, body position with respect to object) of the same object upon verbal instruction [17,29,30]. The results of the present study take this a step further, as participants appeared competent in attending to the dynamical features of the handheld bar independently of those associated with the exoskeleton. Dynamic touch has also proven to be reliable when using movements across different bodily segments (e.g., lower limbs, trunk, and head [30,31]) demonstrating that this perceptual system is capably assembled across different anatomical structures. Here, the integration of the exoskeleton indeed complexified the architecture of the upper limb effector, although one can only speculate as to whether its (non-physiological) components are co-opted into the smart haptic system. Nonetheless, the results of the present study provide evidence that the addition of the exoskeleton does not impede the transmission, or ability to attend to the mechanical stimulus necessary for dynamic touch. 

In certain respects, the findings of this study are encouraging for the ongoing development of exoskeletons as assistive technology for the upper limb. Specifically, this work suggests that the mechanical coupling of the human arm to an exoskeleton would not impair nonvisual perception of handheld tools or utensils in functional tasks. It is also interesting to note that participants tended to use less time for estimations with shorter bars (and less mass). It might thus be conceivable that an exoskeleton with an assistive mode (e.g., antigravity) might indeed facilitate dynamic touch with objects of greater mass. At the same time, caution should be exercised in extrapolating these findings to situations where a robotic exoskeleton intervenes through the course of the user’s movements. Dynamic touch is above all else an active form of perception, dependent upon the motor activity of the user. Moreover, previous studies suggest that efferent copies of intended movements (corollary discharge) are exploited for tuning sensory processing [32]. By modifying the dynamics of the planned exploratory movement, a robotic exoskeleton may consequently degrade nonvisual perception during dynamic touch. This may be less problematic in simple force fields where a user might adapt to the modified environment [33], or in cases where highly sophisticated predictive control is implemented [34]. Conversely, dynamic touch might be particularly challenging when carried out with complex control algorithms such as those which intervene based on intersegmental coordination [35]. Regardless, future studies will need to verify dynamic touch capacities during situations for co-manipulation to ensure that users might effectively perform dextrous tool use activities in collaboration with upper limb exoskeletons. 

Several limitations should be recognized in the current study. Firstly, the size of the experimental cohort was modest (*n* = 12) and the inclusion of a greater number of participants would potentially yield further statistically significant differences (e.g., interaction effects for response times). Kinematic data would also have been interesting to improve understanding of user behaviour. Indeed, it is possible that participants used different exploratory movement strategies with the exoskeleton attached. Following the example of previous studies [36], kinematic data might be used to gauge skill in perceptual sampling during tool use. In addition to this, load cells at the attachment site between the arm and the exoskeleton might have been included in the experimental setup to examine the exchange of forces between the user and the exoskeleton. Finally, the exoskeleton fixed at the level of the upper arm and forearm imposed mechanical consequences through the shoulder and elbow joints. It remains to be seen how exteroception is affected when an exoskeleton is applied directly across articulations of the wrist and hand.

## 5. Conclusions

The fixation of an exoskeleton to the human arm implies local compression to soft tissue structures at the site of attachment, while the mass and inertial consequences of the structure impose additional loads upon the user through the course of their movements. Both of these factors (i.e., soft tissue compression and joint loading) solicit cutaneous and neuromuscular mechanoreceptors which support haptic perception. Here we presented two experiments designed to examine the potential effects of attachment and exoskeleton mass upon participant exteroception. Our results indicated that nonvisual perception of partial length was not adversely affected by mechanical coupling to an exoskeleton fixed to the forearm and upper arm of the user. These findings suggest that the nervous system dissociates mechanical consequences of movement attributable to the handheld object from those attributable to the exoskeleton. To our knowledge, this is the first study which directly examines nonvisual perception of handheld objects when using an upper limb exoskeleton.

It is likely that findings from the present study could be generalized to other forms of dynamic touch. As they are derived in much the same manner as exteroception [17], we anticipate that mechanical coupling with an exoskeleton would have limited consequences upon exproprioception and proexteroception. Extrapolation to surface haptics is somewhat more difficult. However, given that these types of exploratory procedures (e.g., enclosure, lateral motion, contour following, etc) rely more exclusively upon stimulation of cutaneous sensory receptors at the interface with the external environment, they might inherently be less susceptible to perturbations from the structure of the exoskeleton itself. The same might not be true for other types of upper limb assistive technology such as vibrotactile matrices [37].

As an extension of the work presented here, two lines of research might be envisaged. In the first instance, the effects of a mechanical structure being attached to more distal joints of the upper limb should be investigated. In the experiments carried out here, movements of the wrist were unconstrained by the exoskeleton during object handling. Subsequent studies might examine how fixation of a device to the hand or fingers influences nonvisual perception of handheld objects. The second line of research might examine how interaction with robotic control algorithms influences exteroception. In effect, robotic control in human exoskeletons has generally be developed to support somewhat regular prehensile and locomotor function (e.g., reaching, leg swing during gait [5,38]). These forms of assistive control are likely to be less adapted to comparatively irregular exploratory movements which are highly dependent on action-perception coupling [17]. In both cases, kinematic analysis could be employed to examine the exploratory movements as the user seeks to extract the dynamical variables pertinent to dynamic touch, according to the conditions imposed through the interaction with the upper limb exoskeleton.

## Data Availability

Data obtained during this experiment can be provided upon request. Additional details regarding statistical analysis are available in the supplementary materials provided online.

## Supplementary Materials

Additional resources including statistical tables and a video recording of an experimental trial are available at https://doi.org/10.6084/m9.figshare.22302901.v2.
